# Clostridium perfringens Produces an Adhesive Pilus Required for the Pathogenesis of Necrotic Enteritis in Poultry

**DOI:** 10.1128/JB.00578-20

**Published:** 2021-03-08

**Authors:** D. Lepp, Y. Zhou, S. Ojha, I. Mehdizadeh Gohari, J. Carere, C. Yang, J. F. Prescott, J. Gong

**Affiliations:** a Guelph Research and Development Centre, Agriculture and Agri-Food Canada, Guelph, Ontario, Canada; b Department of Pathobiology, University of Guelph, Guelph, Ontario, Canada; c Department of Animal Science, University of Manitoba, Winnipeg, Manitoba, Canada; University of Illinois at Chicago

**Keywords:** *Clostridium perfringens*, necrotic enteritis, chicken, pili, sortase

## Abstract

Clostridium perfringens type G strains cause necrotic enteritis (NE) in poultry, an economically important disease that is a major target of in-feed antibiotics. NE is a multifactorial disease, involving not only the critically important NetB toxin but also additional virulence and virulence-associated factors. We previously identified a C. perfringens chromosomal locus (VR-10B) associated with disease-causing strains that is predicted to encode a sortase-dependent pilus. In the current study, we sought to provide direct evidence for the production of a pilus by C. perfringens and establish its role in NE pathogenesis. Pilus structures in virulent C. perfringens strain CP1 were visualized by transmission electron microscopy (TEM) of immunogold-labeled cells. Filamentous structures were observed extending from the cell surface in wild-type CP1 but not from isogenic pilin-null mutant strains. In addition, immunoblotting of cell surface proteins demonstrated that CP1, but not the null mutant strains, produced a high molecular weight ladder-like pattern characteristic of a pilus polymer. Binding to collagen types I, II, and IV was significantly reduced (Tukey’s test, *P* < 0.01) in all three pilin mutants compared to CP1 and could be specifically blocked by CnaA and FimA antisera, indicating that these pilins participate in adherence. Furthermore, *fimA* and *fimB* null mutants were both severely attenuated in their ability to cause disease in an *in vivo* chicken NE challenge model. Together, these results provide the first direct evidence for the production of a sortase-dependent pilus by C. perfringens and confirm its critical role in NE pathogenesis and collagen binding.

**IMPORTANCE** In necrotic enteritis (NE), an intestinal disease of chickens, Clostridium perfringens cells adhere tightly to damaged intestinal tissue, but the factors involved are not known. We previously discovered a cluster of C. perfringens genes predicted to encode a pilus, a hair-like bacterial surface structure commonly involved in adherence. In the current study, we have directly imaged this pilus using transmission electron microscopy (TEM). We also show that inactivation of the pilus genes stops pilus production, significantly reducing the bacterium's ability to bind collagen and cause disease. Importantly, this is the first direct evidence for the production of a sortase-dependent pilus by C. perfringens, revealing a promising new target for developing therapeutics to combat this economically important disease.

## Abstract (precis)

In necrotic enteritis (NE), an intestinal disease of chickens, Clostridium perfringens cells adhere tightly to damaged intestinal tissue, but the factors involved are not known. We previously discovered a cluster of C. perfringens genes predicted to encode a pilus, a hair-like bacterial surface structure commonly involved in adherence.

## INTRODUCTION

Clostridium perfringens is a facultative anaerobic Gram-positive spore-forming rod, best known for producing an arsenal of over 20 extracellular toxins which contribute to the pathophysiology of a range of histotoxic and of enterotoxic diseases, including gas gangrene and food poisoning in humans, necrotizing enteritis in neonatal farm animals, enterotoxemia in sheep and goats, and necrotic enteritis (NE) in poultry (1). C. perfringens strains are classified into seven toxinotypes (types A to G) based upon the variable production of 6 extracellular toxins, most of whose genes are carried on large conjugative plasmids (2). C. perfringens type G strains, which produce alpha-toxin (CPA) and the necrotic enteritis B-like (NetB) pore-forming toxin, are responsible for causing NE (3–5), an economically important disease of poultry that has been estimated to cost the broiler industry approximately US$6 billion in losses per year (6). NetB is encoded on an ∼42-kb plasmid-borne pathogenicity locus (7) and has been shown to be essential for the development of NE (3), although other virulence or virulence-associated factors are also required (7–9). The pathogenesis of NE is complex and not fully understood, involving out-competition with the existing microbiota and rapid proliferation of type G strains in the small intestine (10), along with production of NetB, which is regulated by the Agr-like quorum sensing system (11), to ultimately produce the characteristic intestinal lesions.

The importance of adherence in bacterial pathogenesis is well established and typically mediated by cell surface structures such as adhesins, flagella, and pili (12). These adhesive cell surface appendages often bind to specific host receptors and thereby define host specificity. In birds suffering from NE, a dense layer of C. perfringens cells can be observed adhering to the small intestinal lesions upon histological examination, suggesting that adherence is a key step in NE pathogenesis (10). Despite this, little is known about the specific C. perfringens adherence factors that are involved in NE or the many other human and animal diseases caused by this pathogen.

We previously identified an ∼9-kb chromosomal locus (VR-10B) associated with NetB-positive strains that consists of seven open reading frames (ORFs), which together are predicted to encode an adhesive sortase-dependent pilus and a two-component system (TCS) (8). This locus is present in 68 to 85% of NetB-positive strains and 0 to 21% of NetB-negative strains but absent from nonpoultry isolates (8, 13, 14).

Sortase-dependent pili are important for the virulence of many Gram-positive pathogens, including Corynebacterium diphtheriae (15), Enterococcus faecalis (16), Streptococcus pyogenes (17), and S. agalactiae (18), but their production has not been previously demonstrated in C. perfringens. The VR-10B pilus-related ORFs are predicted to encode three pilin subunits (CnaA, FimA, and FimB), a signal peptidase, and a class B sortase, which are expressed together in a single polycistronic transcript (13). The VR-10B operon shares sequence similarity and synteny with pilus loci from streptococcal species (8) and, based upon this apparent homology, it is hypothesized that FimA and/or FimB are assembled into the pilus shaft, while CnaA, which possesses a Cna B-type collagen-binding domain, serves as the tip adhesin. It was previously shown that affinity for binding to different collagen types is positively correlated with both virulence and carriage of *cnaA* in various poultry isolates (19, 20). Furthermore, it was also demonstrated that *cnaA*-null mutants of two virulent poultry strains had reduced ability to both bind collagen and cause disease *in vivo*, although differences were observed between strains in collagen type specificity (13). Although sequence analysis predicts that the VR-10B locus confers its functionality through production of a pilus, direct visualization of a pilus in any C. perfringens strain has not been shown.

In the current study, we sought to show the production of a sortase-dependent pilus by C. perfringens, to further elucidate the function of this structure in binding to collagen, and to demonstrate its requirement in the pathogenesis of NE.

## RESULTS

### Generation of C. perfringens pilin-null mutant strains.

To address the hypothesis that the VR-10B locus encodes a functional pilus, null-mutant strains for each of the pilin genes (*cnaA*, *fimA*, and *fimB*) were constructed from the CP1 parent strain by ClosTron targeted mutagenesis (see Fig. S1A in the supplemental material). The presence of the 1.8-kb ClosTron insert at the correct location within each targeted gene was confirmed in the resultant mutant strains (CP1*cnaA*, CP1*fimA*, and CP1*fimB*) by PCR and Sanger sequencing, and Southern blot analysis verified that only a single insertion event had taken place (Fig. S2). Several attempts were made to complement the mutant strains by introducing the individual pilin genes carried on the pJIR750 shuttle vector, preceded by 500 bp of the *cnaA* upstream region to serve as the endogenous promoter, but these were unsuccessful. The complementation was unable to restore pilus production, as determined by immunoblotting (data not shown), despite restoring transcription of the native gene, as demonstrated by reverse transcriptase PCR (RT-PCR) (Fig. S1B). Further attempts were made to construct vectors carrying the full pilus operon, in the event that the lack of pilus production was due to polar effects exerted by the mutagenesis; however, attempts to clone this fragment were also unsuccessful, possibly due to the large insert size.

As an alternative, each pilus mutant was sequenced and compared with the CP1 parent strain to identify off-target mutations that might affect pilus expression. Variants that did not fall within a predicted protein coding region or result in an amino acid change (i.e., synonymous mutations) were disregarded. A total of 41, 28, and 23 SNPs that differed from the CP1 reference genome were identified in the CP1*cnaA*, CP1*fimA*, and CP1*fimB* genomes, respectively (Table S1), while no indels were found. No variants were observed within the VR-10B region, aside from the ClosTron insertion itself. Seven variants were present in multiple strains, and four genes were affected by more than one SNP, resulting in a total of 83 unique SNPs within 79 predicted genes (Table S2). Since the identical variants present in multiple strains more likely originated from a mutation in the reference genome than from independent mutations in the derivative strains, those mutant strains lacking the shared SNPs were inspected further. In all cases, an identical variant was observed in these strains, though at a read coverage below the cutoff threshold, which resulted in the false-negative call. Visual inspection of the read alignments surrounding each SNP also revealed that five were poorly supported by the data, as they were not present in all reads or were also present in the reference reads, and likely represented false positives (data not shown).

### Immunoblotting of CP1 pilin-null mutant strains.

**FIG 1:**
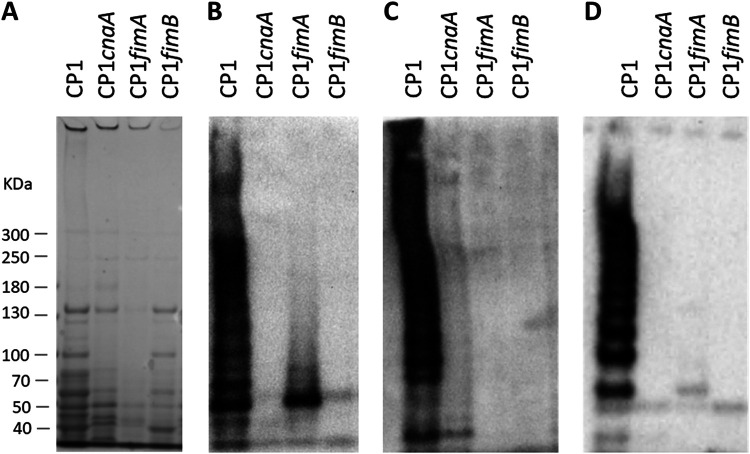
Pilus production in C. perfringens CP1 and isogenic pilin null-mutant strains. Surface-associated protein was extracted from 10 ml of C. perfringens TGY culture (OD_600_ of ∼1) by muramidase treatment and resuspended in 50 μl of sample loading buffer. Extract (5 μl) was separated on a 3 to 8% Tris-acetate gel and immunoblotting was performed using rabbit antiserum (1:200) prepared against each purified pilin protein, followed by goat anti-rabbit IgG AP-conjugated secondary antibody (1:2,000). (A) Coomassie-stained gel. (B) Anti-CnaA. (C) Anti-FimA. (D) Anti-FimB.

When immunoblotting was performed on cell surface proteins extracted from CP1 and the isogenic mutant strains, a high-molecular-weight (HMW) pattern indicative of a polymeric pilus was observed in CP1 but was absent from the mutant strains (Fig. 1). A similar pattern of detection was observed in immunoblots when using antiserum from each of the three pilin subunits.

**FIG 2:**
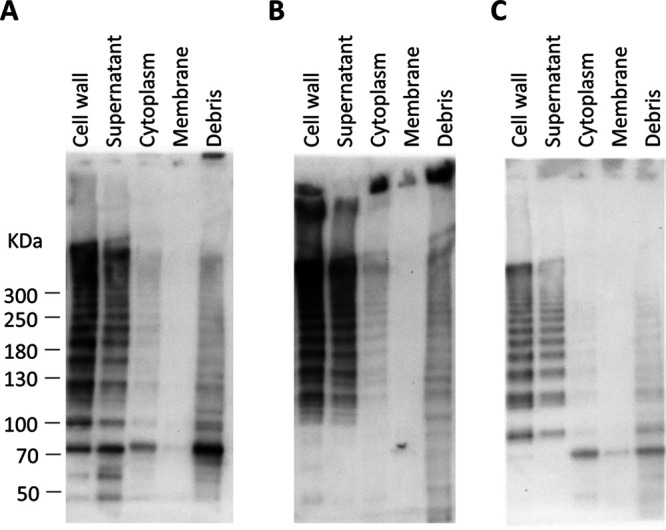
Subcellular localization of pilus proteins in C. perfringens CP1. Total protein was separated by centrifugation and enzymatic treatment into five subcellular fractions and immunoblotting was performed using antiserum against CnaA (A), FimA (B), and FimB (C).

To determine the subcellular location of the pilus, different protein fractions, including cytoplasmic, secreted, cell wall, and membrane-associated, were prepared from C. perfringens CP1 cultures grown in tryptone-glucose-yeast (TGY) broth and visualized by immunoblotting with CnaA, FimA, and FimB antisera. As expected, pilus protein was found predominantly in the cell wall and, to a lesser extent, in secreted fractions (Fig. 2).

Based on the presence of a collagen-binding domain, CnaA is predicted to be the tip adhesin, while FimA and/or FimB may serve as the base or backbone pilins. To infer the putative functions of the individual pilin subunits, densitometric analysis was carried out on the immunoblots produced from each primary antibody. This analysis revealed that each band larger than 121 kDa (the theoretical size of a pilus composed of each single subunit) incremented by ∼32 kDa, the predicted size of FimA following removal of the signal peptide and cell wall sorting signal (CWSS) (data not shown). Thus, each band in the “ladder” is indicative of a pilus with one additional FimA subunit. This observed banding pattern is consistent with an assembled pilus composed of a single subunit each of FimB and CnaA, and multiple subunits of FimA (Fig. S2). Further evidence for the proposed structure is provided by the increased intensity of the anti-FimA immunoblots in proportion to band size, suggesting that multiple copies of FimA accumulate in the pilus multimer as it is assembled (Fig. 2B). The anti-CnaA and anti-FimB immunoblots, on the other hand, did not exhibit this relationship between band size and intensity.

### Visualization of pilus structures by TEM immunogold labeling.

**FIG 3:**
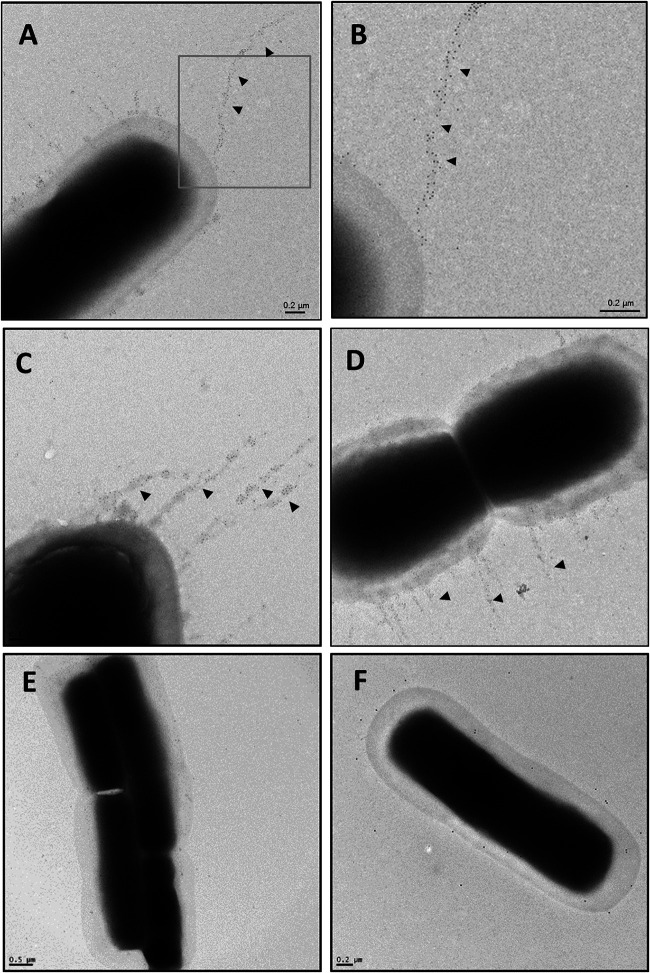
Transmission electron microscopy of C. perfringens CP1 and isogenic pilin null mutants immunogold labeled with pilin antiserum. C. perfringens cultures were grown to mid-log-phase in TGY broth and allowed to adhere to Formvar carbon-coated grids. Grids were washed three times with PBS, blocked in 0.1% gelatin, and sequentially incubated with rabbit antipilin primary antibody (1:20), followed by 6 nm colloidal gold-conjugated goat anti-rabbit secondary antibody (1:20). Bacterial cells were negatively stained with uranyl acetate and examined under a Philips CM10 TEM. (A to D) CP1 plus anti-FimA; (B) inset of panel A; (E) CP1*fimA* plus anti-FimA; (F) CP1*fimB* plus anti-FimB.

To directly visualize pili expressed on the bacterial cell surface, transmission electron microscopy (TEM) was performed on immunogold-labeled CP1 and the isogenic mutant CP1*fimA* and CP1*fimB* cells. When CP1 cells were labeled using FimA antiserum, filamentous structures decorated with gold particles were observed extending >1 μm from the cell surface. The gold particles were distributed throughout the length of the pilus, further indicating that FimA serves as the backbone pilin. In contrast, these structures were absent from both CP1*fimA* and CP1*fimB* cells labeled with FimA and FimB antiserum, respectively (Fig. 3E and F).

### Binding to collagen is blocked by pilin-specific antisera.

**FIG 4:**
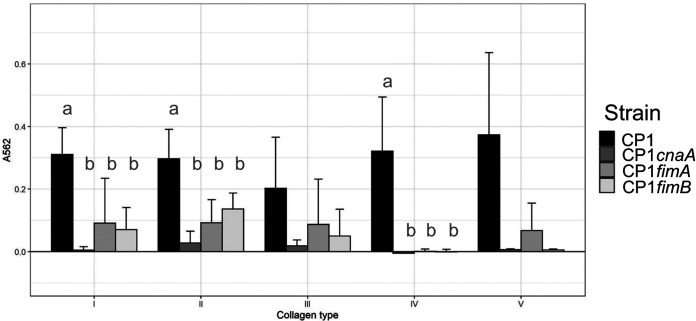
Adherence of C. perfringens CP1 and isogenic pilin null mutants to different collagen types. Ninety-six-well plates were coated overnight with collagen types I to V and C. perfringens cultures, grown overnight on BHI plates and suspended in PBS to an OD_600_ of ∼1, were allowed to adhere for 2.5 h and then stained with 0.5% (wt/vol) crystal violet solution. Following 3 washes with PBS, wells were destained with ethanol-acetone (1:1) and absorbance was measured at 562 nm. Letters indicate significantly different groups (*P* < 0.05; Tukey’s test). Error bars represent standard deviation (SD).

Collagen-binding assays using collagen types I to V were performed with CP1 and the three pilin mutant strains to investigate the collagen specificity of the CP1 pilus. CP1 adhered to all five of the collagen types examined. In contrast, binding to collagen types I, II, and IV was significantly reduced in all three pilin mutants (Tukey’s test, *P* < 0.05) and a similar, though not significant, trend was observed for types III and V (Fig. 4).

**FIG 5:**
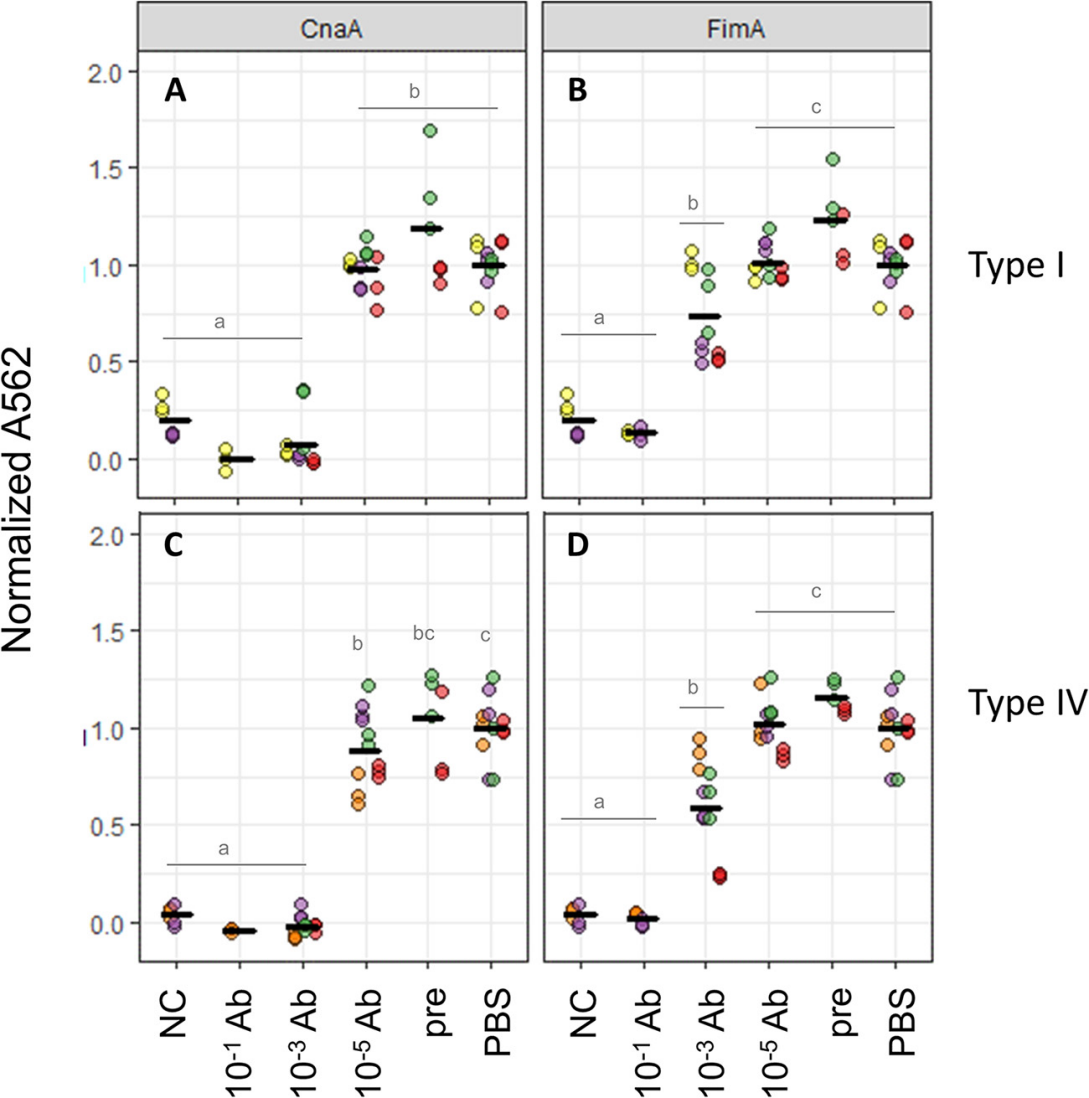
Blocking of C. perfringens CP1 collagen binding by antipilus antibodies. Ninety-six-well plates were coated with collagen types I (A to C) or IV (D to F) and CP1 cells were incubated with dilutions of rabbit antiserum against pilins CnaA (A and D), FimA (B and E), or FimB (C and F). *A*_562_ values were normalized to average *A*_562_ from CP1 incubated with PBS. NC, no collagen; PBS, CP1 incubated with PBS; pre, CP1 incubated with preimmune serum (10^−3^ dilution). Colors indicate data points from different assays and horizontal bars represent means. Letters indicate significantly different groups (*P* < 0.05; Tukey’s test).

To verify that the observed collagen binding could be specifically attributed to the pilus, CP1 cultures were preincubated with different dilutions of pilin antisera and assayed for binding to collagen types I and IV. Antisera against both CnaA and FimA were able to significantly reduce CP1 adherence to both collagen types at dilutions of 10^−1^ and 10^−3^ in a dose-dependent manner (Fig. 5). As a negative control, cultures preincubated with 10^−3^ preimmune serum showed no inhibitory effect on collagen binding of CP1. These findings further support the specific role of this pilus in binding to both collagen types I and IV, and indicate that both CnaA and FimA contribute to this activity.

### C. perfringens CP1 pilin-null mutants do not cause NE *in vivo*.

**FIG 6:**
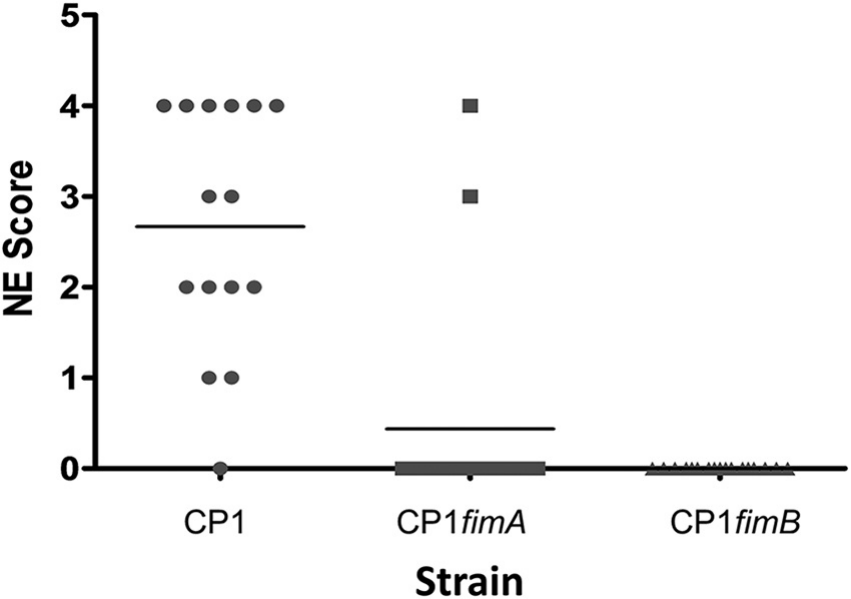
NE lesion scores of chickens experimentally infected with wild-type CP1 or pilin null mutants. Groups of 17 male Ross 708 birds were challenged in-feed with C. perfringens CP1, CP1*fim*A, or CP1*fim*B culture twice daily for 2 days. Birds were sacrificed on day 29 and scored for intestinal lesions.

To determine the role of the pilus in the ability of C. perfringens CP1 to cause NE, groups of birds were experimentally infected with wild-type CP1 or with CP1*fimA* or CP1*fimB* isogenic mutant strains. Virulence was severely attenuated in CP1*fimA* and CP1*fimB* mutants, which were both unable to cause significant disease *in vivo* (average NE scores of 0.43 and 0, respectively, versus the WT control score of 2.6; *P* < 0.005, Fisher’s least significant difference [LSD]), indicating a requirement for the pilus in NE pathogenesis (Fig. 6).

## DISCUSSION

Sortase-dependent pili are produced by a number of Gram-positive pathogens and are frequently involved in virulence-related processes, such as host cell adhesion, during colonization and biofilm formation (21). In the present study, we sought to provide direct evidence for the production of a sortase-dependent pilus by C. perfringens encoded by the VR-10B locus, and to further elucidate its role in adherence and NE pathogenesis. Site-directed mutagenesis of each of the three predicted pilin subunits found in the VR-10B locus resulted in complete loss of pilus production, as demonstrated by both immunoblotting and TEM immunogold labeling. While attempts to complement the pilin null-mutant strains were unsuccessful, several lines of evidence indicate that this phenotype did not arise from nonspecific mutations. First, Southern blot analysis and PCR verified the presence of a single chromosomal insert only within the targeted gene in each of the isogenic mutants. Second, whole-genome sequencing of the three mutants did not identify consistent variants among the three mutants that could explain their shared phenotype. The vast majority of the identified SNPs resulted in missense mutations, which do not typically confer significant fitness effects, although 10 variants gave rise to premature stop codons (nonsense mutations) that are more likely to be deleterious. These latter mutations fell within genes encoding an iron-sulfur cluster carrier protein, transcription elongation factor GreA, putative glycosyltransferase EpsD, dTDP-4-dehydrorhamnose 32C5-epimerase, alpha-galactosidase AgaA, heme oxygenase 1, chromosome partition protein Smc, and three hypothetical proteins but not within the *cnaA*, *fimA*, and *fimB* coding sequences. While it cannot be ruled out that these nonsense mutations impacted fitness, none were common among the three strains and therefore could not be responsible for their shared phenotype. Finally, the three pilin mutants represent independent mutations of the same genetic locus, as they target different regions within the same polycistronic transcript. The evaluation of independent mutants is generally an acceptable alternative to complementation, given the improbability of generating identical phenotypes due to spurious nonspecific mutations.

Collagen-binding assays revealed broad binding of wild-type CP1 to collagen types I to V, while binding to collagens I, II, and IV was significantly reduced in all three pilin null mutants. A previous study that examined two virulent C. perfringens poultry isolates, EHE-NE18 and 56, and their *cnaA* null-mutant derivatives, reported similar results for type IV collagen but saw minimal binding of either strain to type I collagen (13). Furthermore, *cnaA*-null mutants of both strains were found to retain their ability to bind type II and III collagens, leading to the conclusion that the pilus does not specificity bind to collagen types I, II or III. In the present study, however, binding of CP1 to collagens I and IV was also blocked by antiserum against both CnaA and FimA, strongly indicating that the observed collagen binding is specifically mediated by the pilus. Genetic variability in the pilus genes cannot account for the apparent differences in binding affinity between the two studies, since CP1 and EHE-NE18 share identical protein sequences for all three pilins (the pilin sequences of strain 56 are not publicly available for comparison). Whether the observed differences were a result of differences in the binding assay factors remains to be clarified. Our antiserum blocking results also suggest that both the tip and backbone pilin contribute to collagen binding, while the base pilin does not. Previous reports of other Gram-positive pili have also shown that pilins other than the tip adhesin may have adherent abilities and participate in host cell binding (22, 23).

The ability of both CP1*fimA* and CP1*fimB* null-mutant strains to cause NE was severely attenuated *in vivo* compared to the wild-type parent strain, consistent with the findings reported by Wade et al. using *cnaA* null-mutant derivatives of strains EHE-NE18 and 56 (13). The avirulent phenotype shared among pilin-null mutants of geographically unrelated strains suggests the pilus plays a critical role in causing NE, possibly via adherence to host collagen or related matrix molecules. This interaction has yet to be demonstrated *in vivo*, nor has it been determined at what specific stage it occurs, whether during initial colonization or later in the disease process following lesion development. Collagen is a component of the extracellular matrix (ECM), implying that epithelial damage must first be incurred to expose ECM molecules for binding. Further studies are required to elucidate the sequence of these events during the disease process. It should also be noted that ∼25% of NetB-positive strains do not carry the VR-10B locus (8, 13) and must therefore employ alternative adhesive strategies during pathogenesis, possibly through the expression of additional adhesive factors yet to be identified.

Gram-positive sortase-dependent pili are heteropolymers of pilin proteins, typically composed of a major backbone pilin (BP) that forms the shaft and one or two minor ancillary pilins (AP) located at the base and/or tip (24–26). Pilins are substrates for sortase transpeptidase enzymes, which catalyze the covalent linkage of pilin subunits at the cell surface via isopeptide bonds. Sortases are categorized into classes A to F (27), with each class generally having specificity for a different substrate based upon recognition of its C-terminal pentapeptide cell wall sorting signal (CWSS). Pilus genetic islands typically encode one or more class C sortases, which function to both initiate and extend the polymerization of tip and backbone pilins through recognition of their cognate CWSSs. The incorporation of the base pilin is then performed by a class A housekeeping sortase, which also anchors the completed structure onto the peptidoglycan layer, thereby terminating pilus assembly. This canonical system, as first elaborated in C. diphtheriae (28, 29), has since been identified in many other Gram-positive species, with several variations described. For instance, the Bacillus cereus pilus lacks a base pilin and terminates elongation stochastically (30), whereas the S. pneumoniae pilus-islet 2 encodes a single pilin that functions as both the backbone and tip adhesin (23). Some pilus islands also encode multiple sortase C enzymes, which may have redundant functionality (31).

**FIG 7:**
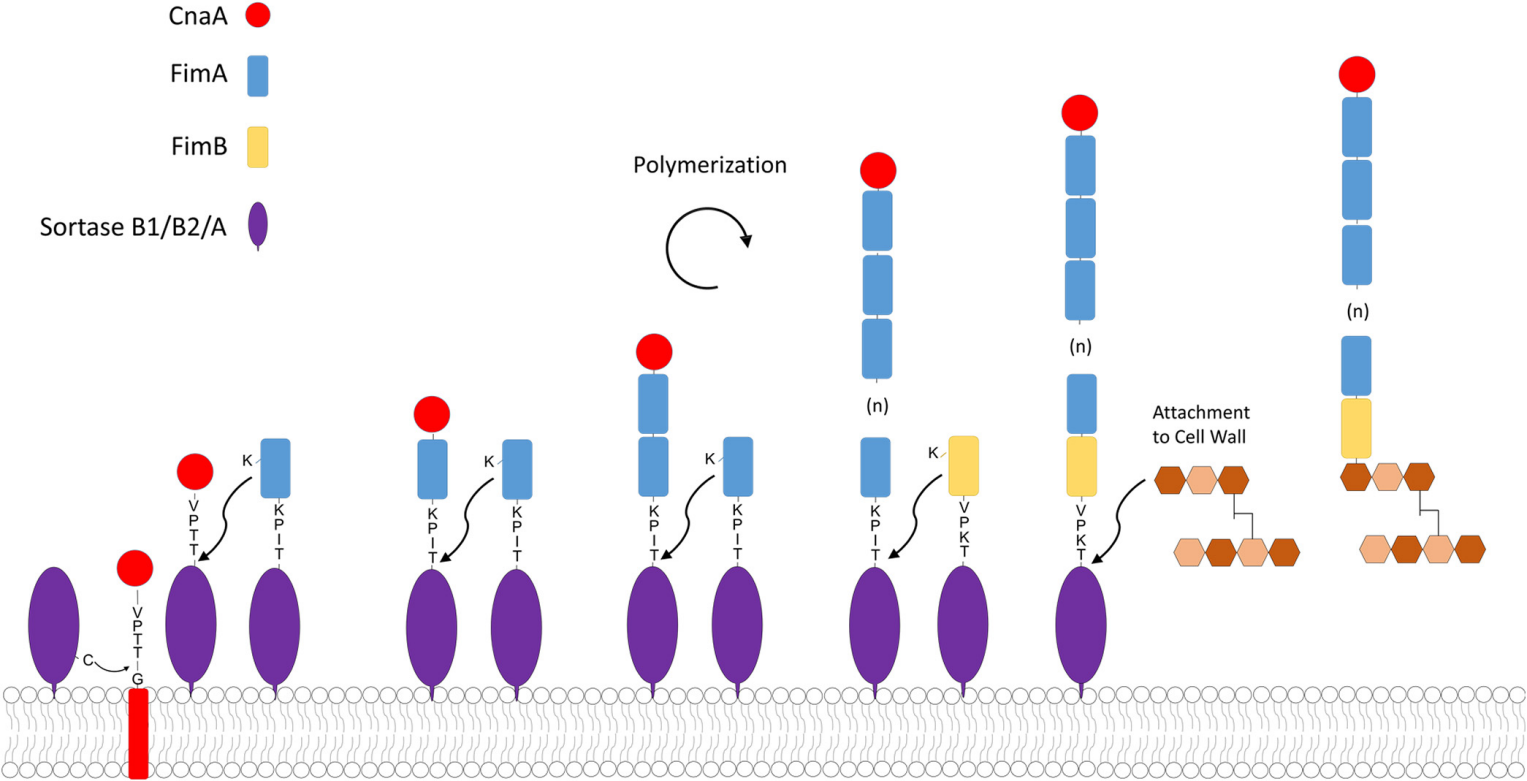
Diagram of proposed NE pilus assembly mechanism. The proposed assembly is initiated by transfer of the CnaA tip pilin onto the sortase B1 or B2 enzyme and subsequent polymerization of FimA to form the pilus backbone. FimB is finally linked to FimA by sortase B1, B2, or the housekeeping sortase A and the completed structure is transferred onto the peptidoglycan layer. The amino acid sequences of the predicted cell wall sorting signals (CWSS) and cleavage site are indicated for each pilin.

The VR-10B locus contains three pilin genes predicted to encode the backbone, tip, and base pilins, similar to the canonical C. diphtheriae sortase-dependent pilus operon. CnaA possesses a collagen-binding domain and is thus the predicted tip adhesin, leaving FimA and FimB as the most likely candidates for backbone pilin. Immunoblotting of CP1 surface proteins with pilin antisera produced bands with incremental sizes corresponding to that of FimA. Furthermore, TEM immunogold-labeled images using FimA antiserum revealed gold particles extending along the length of the pilus fiber. Taken together, these results indicate that FimA serves as the major backbone pilin in the NE pilus (Fig. 7). Interestingly, VR-10B encodes a class B sortase, whose gene is denoted in Fig. S1A in the supplemental material as *srtB1*, commonly involved in the anchoring of iron uptake proteins, instead of a class C sortase as is typically found in other Gram-positive pilus islands. Although unusual, the S. pyogenes Spy128 pilus also employs a class B sortase for pilus assembly (32). Additionally, the VR-10B FimB putative base pilin contains a “sortase B cell surface sorting signal” domain (TIGRFAM TIGR03063), suggesting that it is a substrate for a sortase B enzyme and not the housekeeping class A sortase. A second putative sortase B gene (CPE0513 in C. perfringens strain 13; denoted in Fig. S1A as *srtB2*), which is highly conserved among C. perfringens genomes, is found directly upstream of both the VR-10A and -10B alleles but in the opposite orientation. Further studies are required to determine what role, if any, is played by both sortase B2 and the housekeeping sortase A in pilus assembly. We suggest that the pilus encoded by the VR-10B operon is assembled as shown in Fig. 7.

One other putative sortase-dependent pilus island (*Cpp*) was previously identified in C. perfringens (33), and it was recently demonstrated that polymerization of the major pilin is catalyzed by an adjacent class C sortase, although confirmation by TEM was not performed (34). In addition, most C. perfringens strains also produce a type IV pilus involved in gliding motility (35), which differs fundamentally from sortase-dependent pili in structure, function, and assembly mechanism. To distinguish the pilus characterized in the present study from other C. perfringens pili, we propose the designation “NE pilus.” Unlike the NE pilus, which is found predominantly in NE-associated C. perfringens strains, the putative *Cpp* pilus gene is widely distributed among the genomes of distantly related strains isolated from various hosts, and it is not yet clear if it participates in adherence to host factors. It is possible that additional C. perfringens pilus islands exist that are limited to specific strain types, as is found with other Gram-positive pathogens.

Most C. perfringens strains originating from either healthy birds or nonavian sources carry an alternate region (VR-10A) at the same chromosomal location as VR-10B, the function of which is unknown (8, 13, 14). While VR-10A does not appear to possess the necessary pilin and sortase genes required for the production of a pilus, it encodes a putative cell surface protein that contains a vWFA domain, named for the human vWFA that is involved in collagen binding (36). This domain is present in several tip adhesin pilins (15, 24, 37–39) and has been specifically implicated in binding of S. agalactiae to epithelial cells (40). It remains to be determined if this genomic region also plays a role in adherence or contributes to the pathogenesis of other C. perfringens-caused diseases, although its distribution across a diverse set of strains implies that, unlike the poultry- and NE-associated VR-10B locus, it is not a specific determinant of host range or pathotype. Both VR-10A and B encode a TCS, although the putative response regulator and sensor kinase protein sequences between the two variants share only ∼70% and 83% identity, respectively, suggesting that they may respond to different environmental stimuli and/or recognize different target promoter binding sites (8). Additional studies are needed to determine if the VR-10B TCS plays a role in regulating pilus production.

A third variant of this locus, VR-10C, was recently identified by comparative genomics and consists of only the conserved flanking genes, apparently lacking an insertion (14). This variant was found in three poultry isolates within nonpathogenic clade 2, whereas VR-10B was present exclusively in poultry isolates of pathogenic clades 1A and 1B. These findings provide further evidence that VR-10B is reserved to virulent poultry isolates and, as such, may define the host range of these strains.

In the present study, we have provided the first direct evidence for the production of a sortase-dependent pilus by C. perfringens. These results further establish the essential role of the NE pilus in pathogenesis and confirm previous findings that the VR-10B locus is involved in binding to collagen but suggest a broader specificity for collagen types I, II and IV. Given the apparent importance of this pilus in virulence, it represents a novel potential therapeutic target for the control of NE in broilers but points also to the potential existence of additional cell surface adhesins or pili that contribute to the virulence of other C. perfringens pathotypes. Further studies are needed to shed light on the regulation of this pilus and define more precisely its role in NE pathogenesis, as well as the alternative processes used by virulent strains that lack this locus.

## MATERIALS AND METHODS

### Bacterial strains and culture conditions.

C. perfringens strain CP1 used in this study was a field isolate from an NE case in Ontario, Canada (41). Bacterial culture media used throughout this study included tryptone-glucose-yeast (TGY; 3% tryptone, 2% glucose, 1% yeast extract), fluid thioglycolate (FTG; Difco, Sparks, MD), brain heart infusion (BHI) (Thermo Fisher, Burlington, ON, Canada), and cooked meat medium (CMM; Difco), supplemented with 34 μg ml^−1^ chloramphenicol (Sigma-Aldrich, St. Louis, MO) and 10 μg ml^−1^ erythromycin (Thermo Fisher) as required. Escherichia coli strain Stellar (TaKaRa Bio, Mountain View, CA) and E. coli strain BL21(DE3) (Sigma-Aldrich) competent cells were used for cloning in Luria-Bertani (LB) broth or agar (Difco), supplemented with 34 μg ml^−1^ chloramphenicol (Sigma-Aldrich) as required.

### Generation of C. perfringens mutants.

The ClosTron system was used to insertionally inactivate *cnaA*, *fimA*, and *fimB* in CP1 as described previously (42, 43). ClosTron intron-targeting regions were designed to insert at the following gene positions using the Perutka algorithm implemented at www.clostron.com: 183 bp of the *cnaA* sense strand, 231 bp of the *fimA* sense strand, and 273 bp of the *fimB* sense strand. The intron-targeting regions were synthesized and cloned into ClosTron plasmid pMTL007C-E2 by DNA 2.0 (Menlo Park, CA) and named pMTL007C-E2::572-183s, pMTL007C-E2::574-231s, and pMTL007C-E2::576-273s, respectively. The resultant plasmids were separately electroporated into CP1 as described previously (11) to generate strains CP1 *cnaA183*::*ermB*, CP1 *fimA231*::*ermB*, and CP1 *fimB273*::*ermB*, here referred to, respectively, as CP1*cnaA*, CP1*fimA*, and CP1*fimB*. To verify that the intron had inserted into the expected location, PCR was performed with the primer pairs cnaA-F1/R1 for *cnaA*, fimA-F1/R1 for *fimA*, and fimB-F1/R1 for *fimB* (Table 1), which flank the ClosTron insertion site of each gene. Southern blot analysis was performed on the CP1 wild type and isogenic mutants genomic DNA digested with DraI using a digoxigenin-labeled probe specific for the ClosTron insert using the DIG Luminescent detection kit (Roche, Laval, QC, Canada), as previously described (11).

**TABLE 1:** List of primers used in this study

Primer name	Purpose	Sequence (5′–3′)
ErmF	Mutant verification	GATATTCACCGAACACTAGG
ErmR	Mutant verification	TTACCTGTTCCAATTTCGTA
CT_probe-F1	Southern blot probe synthesis	ACAACTTAATTATACCCACT
CT_probe-R1	Southern blot probe synthesis	CTTGTGTTTATGAATCACG
cnaA-R1	Mutant verification, RT-PCR	CCTTGCTTGGATTCACCAGT
cnaA-F1	Mutant verification, RT-PCR	AAAATAAATAAAAAAATTTTTAGCATGCTATTTATGG
fimA-R1	Mutant verification	CCACCTACTCCCTCATTCGT
fimA-F1	Mutant verification	ATAAACAAGAAAAAATTAAGTGC
fimB-F1	Mutant verification	GAAACAAAGAAAATAAGAAACAAAATCCTTATGGC
fimB-R1	Mutant verification	TTCTTTTTTTCTCTTTTTATATTCTTTCCATTTC
cnaA-F2	RT-PCR	GGTGGATGGGCAACATTTAC

### Reverse transcriptase PCR.

Total RNA was isolated from 1 ml of bacterial culture grown anaerobically in TGY medium to optical density at 600 nm (OD_600_) of ∼1 using the Ambion RiboPure-Bacteria (Thermo Fisher) kit, according to the manufacturer’s instructions. Total RNA (10 μg) was treated with Turbo DNase (Thermo Fisher) and cDNA was generated from 1 μg of RNA using the High Capacity RNA-to-cDNA kit (Thermo Fisher). Reactions were set up in duplicate with (RT) or without (no RT) reverse transcriptase added. PCR was performed using a 1/10 dilution of cDNA as the template, and either Hot Start *Taq* 2× MasterMix (New England BioLabs, Ipswitch, MA) or 2× CloneAmp HiFi PCR Premix (TaKaRa Bio USA, Mountain View, CA).

### Genome sequencing and variant analysis.

Genomic DNA was extracted from each strain with the Gentra PureGene yeast/bacteria kit (Qiagen) according to the manufacturer’s instructions, and sequencing libraries were prepared from 1 ng genomic DNA using the Nextera XT kit (Illumina). Each strain was prepared in duplicate and libraries were sequenced in separate runs on a MiSeq instrument (Illumina) using a 600-cycle v3 reagent kit. The resulting 300-bp paired-end reads were quality-filtered with Trimmomatic (0.39) (44) to remove Nextera adapter sequences and low-quality bases using parameters “LEADING:30 TRAILING:30 SLIDINGWINDOW:4:30 MINLEN:200.” The CP1 quality-filtered reads were *de novo* assembled with SPAdes (3.13.1) (45) and annotated with Prokka (1.14.0) (46) for use as a reference genome in variant analysis. Single-nucleotide polymorphisms (SNPs) and insertion/deletions (indels) between CP1 and mutant strains were identified with GATK (4.1.8.1) (47) based on GATK best practices. Briefly, reads were mapped to the CP1 reference genome with bwa mem (0.7.17-r1188) and duplicate reads identified with PicardTools (2.23.4-0) MarkDuplicates. Variants were called with the GATK HaplotypeCaller and sample genotypes assigned with GenotypeGCVFs. The resulting SNPs and indels were quality filtered separately with GATK VariantFiltration using the parameters -filter “QD < 2.0” -filter “QUAL < 40.0” -filter “SOR > 3.0” -filter “DP < 10” -filter “FS > 60.0” -filter “MQ < 40.0” -filter “MQRankSum < -12.5” -filter “ReadPosRankSum < -8.0” and -filter “QD < 2.0” -filter “QUAL < 40.0” -filter -filter “DP < 10” -filter “FS > 200.0” -filter “ReadPosRankSum < -20.0,” respectively. Variants were annotated with SNPEff (5.0.0) (48) and those annotated as “high” or “moderate” impact and conserved between replicate genomes were visualized and further inspected in the Integrated Genome Browser (IGV; 2.8.9) (49).

### Production of recombinant proteins and pilin-specific rabbit antisera.

Six-histidine-tagged recombinant proteins for the three pilin subunits (CnaA, FimA, and FimB), excluding the predicted N-terminal signal peptides and C-terminal transmembrane domains, were expressed in E. coli BL21(DE3) cells and purified with an AKTAprime plus system (GE Healthcare, Montreal, QC, Canada) under native conditions on a HisTrap FF crude column (GE Healthcare) as previously described (50). Recombinant protein (∼4 mg) of each pilin was delivered to Cedarlane Laboratories (Burlington, ON, Canada) for polyclonal antibody production in rabbits, for use in immunoblotting and immunogold labeling experiments.

### Immunogold labeling and transmission electron microscopy.

Immunogold labeling of C. perfringens cells and transmission electron microscopy (TEM) was performed essentially as described with some modifications (51). Briefly, C. perfringens cultures were grown overnight anaerobically in FTG medium, subcultured into 5 ml of TGY medium, and grown for 3 h. Mutant cultures were supplemented with 10 μg ml^−1^ erythromycin. Mid-log-phase cultures (1 ml) were centrifuged at 6,000 × *g* for 30 s, washed once in 0.1 M NaCl, and gently resuspended in 1 ml phosphate-buffered saline (PBS). Each culture was allowed to adhere to Formvar carbon-coated grids by floating the grid, carbon side down, on a drop of bacterial suspension for 30 min. Grids were similarly washed three times in PBS containing 1% bovine serum albumin (PBSB) for 30 s and blotting on 3 mm filter paper. Grids were transferred to PBS containing 0.1% gelatin and blocked for 1 h, washed three times with PBSB, and then incubated for 1 h with rabbit anti-FimA primary antibody (1:20) in PBSB. Following three washes in PBSB, grids were incubated for 1 h with 6-nm colloidal gold-conjugated goat anti-rabbit secondary Ab (Cedarlane), and finally washed five times with sterile water. Cells were negatively stained with uranyl acetate and examined under a Philips CM10 TEM in bright-field mode.

### Extraction of C. perfringens protein fractions.

Total surface proteins were extracted from C. perfringens strains essentially as described by Chang et al. (2013) (51). Strains were grown overnight in TGY medium anaerobically at 37°C, subcultured 1:100 into 10 ml TGY medium and grown to an OD_600_ of ∼1. Cells were pelleted at 6,000 × *g* for 5 min and 1 ml of supernatant was collected for the secreted fraction. The bacterial pellet was washed once in SMM buffer, pH 6.8 (0.5 M sucrose, 10 mM MgCl_2_, 10 mM maleate), and resuspended in 1 ml SMM buffer, to which was added 60 μl of 5U/μl of mutanolysin (Sigma) in muramidase buffer (2 mM acetic acid, 48 mM sodium acetate) and 10 μl of 0.1 M phenylmethylsulfonyl fluoride (PMSF) (Sigma). Following ∼4 h of incubation at 37°C with constant rotation, protoplasts were pelleted at 20,000 × *g* for 5 min, and the supernatant fraction containing cell wall proteins was removed. The pelleted protoplasts were resuspended in 200 μl SMM and lysed by three freeze-thaw cycles. The membrane fraction was recovered by centrifugation at 100,000 × *g* for 30 min at 4°C, and resuspended in 200 μl SMM, while 1 ml of supernatant was collected for the cytoplasmic fraction. The secreted, cell wall, and cytoplasmic fractions were precipitated by addition of 81 μl 100% (wt/vol) trichloroacetic acid (TCA) (Sigma) per ml and incubation at 4°C overnight. Following centrifugation at 20,000 × *g* at 4°C for 20 min, the protein pellet was washed with acetone and slowly resuspended in 50 μl sample loading buffer (62.5 mM Tris-HCl [pH 6.8], 2% SDS, 20% glycerol, 4% β-mercaptoethanol, 3 M urea, 0.01% bromophenol blue) at room temperature for at least 15 min.

### Immunoblotting and image analysis.

Surface protein extracts (5 μl) were loaded onto a Novex NuPAGE 3 to 8% Tris-acetate gel (Fisher Scientific) and electrophoresed at 150V for 1 h. One gel was used for staining with Biosafe Coomassie stain (Bio-Rad, Mississauga, ON, Canada) and replicate gels were transferred onto polyvinylidene difluoride (PVDF) membranes at 30V overnight at 4°C in 1× transfer buffer (48 mM Tris, 39 mM glycine, 20% methanol, 0.1% SDS). Chemiluminescent detection was performed with the WesternBreeze chemiluminescent kit (Thermo Fisher) according to the manufacturer’s instructions, using rabbit antiserum (1:200) against CnaA, FimA, or FimB as the primary antibody and a goat anti-rabbit IgG alkaline phosphatase (AP)-conjugated secondary antibody (1:2,000) (Cedarlane). Immunoblots were imaged on a Bio-Rad GelDoc XR system using Image Lab software. Lanes and bands were detected automatically by the software and confirmed before analysis. The molecular weights of each band that could be interpolated based on the molecular weight marker were exported for further analysis.

### Collagen binding and antibody blocking assays.

Bacterial adhesion to collagen types I through V (Sigma) was assayed essentially as described previously with minor modifications (20). Collagens assayed included type I from rat tail (Sigma), type II from chicken sternal cartilage (Sigma), type III from human placenta (Sigma), type IV from human placenta (Sigma), and type V from human placenta (Sigma). C. perfringens strains were grown overnight anaerobically at 37°C on blood agar plates and subcultured in BHI medium to an OD_600_ of ∼0.8. A 100-μl aliquot was spread onto a BHI plate and incubated overnight anaerobically at 37°C and the overnight growth was harvested from the surface with PBS. The bacterial suspension was pelleted by centrifugation at 5,000 × *g* for 2 min, washed with PBS, and adjusted to an OD_600_ of 1 with PBS. Nunclon Delta Surface 96-well plates (Thermo Fisher) were coated with 50 μl of collagen (1 mg/ml in PBS) overnight at 4°C and wells were blocked with 200 μl of PBS plus 0.5% (wt/vol) bovine serum albumin (BSA) for 2 h at 4°C and then washed three times with 100 μl of PBS. Bacterial suspensions (50 μl) were added to each well and incubated at room temperature for 2.5 h with gentle shaking, and then wells were washed three times with 100 μl of PBS and air dried. Wells were stained with 0.5% (wt/vol) crystal violet for 5 min, washed three times with 100 μl of PBS, and then destained with 50 μl of 1:1 (vol/vol) ethanol-acetone. Absorbance was measured at 562 nm with a BioTek plate reader. Wells without collagen were used as blank controls, and wells coated with collagen but without added culture served as negative controls. Blank values were subtracted from all absorbance values. All assays were repeated three times, with each assay consisting of triplicate wells. The binding values were averaged from three assays with the exception of collagen V, which was repeated twice.

For antibody blocking assays, cultures were first incubated for 20 min with different dilutions of rabbit antiserum against CnaA, FimA, and FimB before adding to the collagen-coated wells. The binding assay was then continued as described above.

### Chicken NE challenge trial.

Experiments with chickens and conditions for their use were approved by the University of Guelph Animal Care Committee in accordance with the Canadian Council on Animal Care’s Guidelines. Commercial one-day-old male Ross 708 broiler chickens (Bonnie’s Chick Hatchery, Elmira, ON, Canada) were randomly divided into three experimental groups (*n* = 16 to 17 per group) and raised in separate floor pens. The chickens were fed an antibiotic-free broiler starter ration containing 20% protein. For the experimental infection (challenge) of birds, wild-type CP1, CP1*fimA*, and CP1*fimB* were grown in CMM for 24 h at 37°C under anaerobic conditions and then inoculated at 3% (vol/vol) into fresh CMM. Prior to each challenge day, the CMM culture was used to inoculate FTG medium and incubated overnight at 37°C. Birds were fasted for 24 h and then switched to an antibiotic-free turkey starter ration (28% protein) containing C. perfringens culture at days 26 and 27. The infected ration was prepared daily in the morning and afternoon by mixing with C. perfringens FTG culture, grown for 15 h and 24 h, respectively, at a 2:1 (v/w) ratio of the culture to feed. On day 29, chickens were euthanized with carbon dioxide, and at necropsy the small intestine was examined for grossly visible lesions. NE lesion severity was scored blindly from 0 to 6 using the system described by Keyburn et al. (3).

### Statistical analyses.

Collagen binding data are presented as means of experimental replicates ± SD, with each experimental replicate the average of triplicate wells. Comparison between multiple groups was performed using ANOVA followed by pairwise comparisons with Tukey’s *post hoc* test, where a *P* value of ≤0.05 was considered significant.

## Supplementary Material

Supplemental file 1
