# Supplementary material for: Clostridium perfringens Produces an Adhesive Pilus Required for the Pathogenesis of Necrotic Enteritis in Poultry
Source: J Bacteriol. 2021 Mar 8;203(7):e00578-20. doi: 10.1128/JB.00578-20 (PMC8088525; doi:10.1128/JB.00578-20)
Supplement: Supplemental file 1 [file JB.00578-20-s0001.pdf]

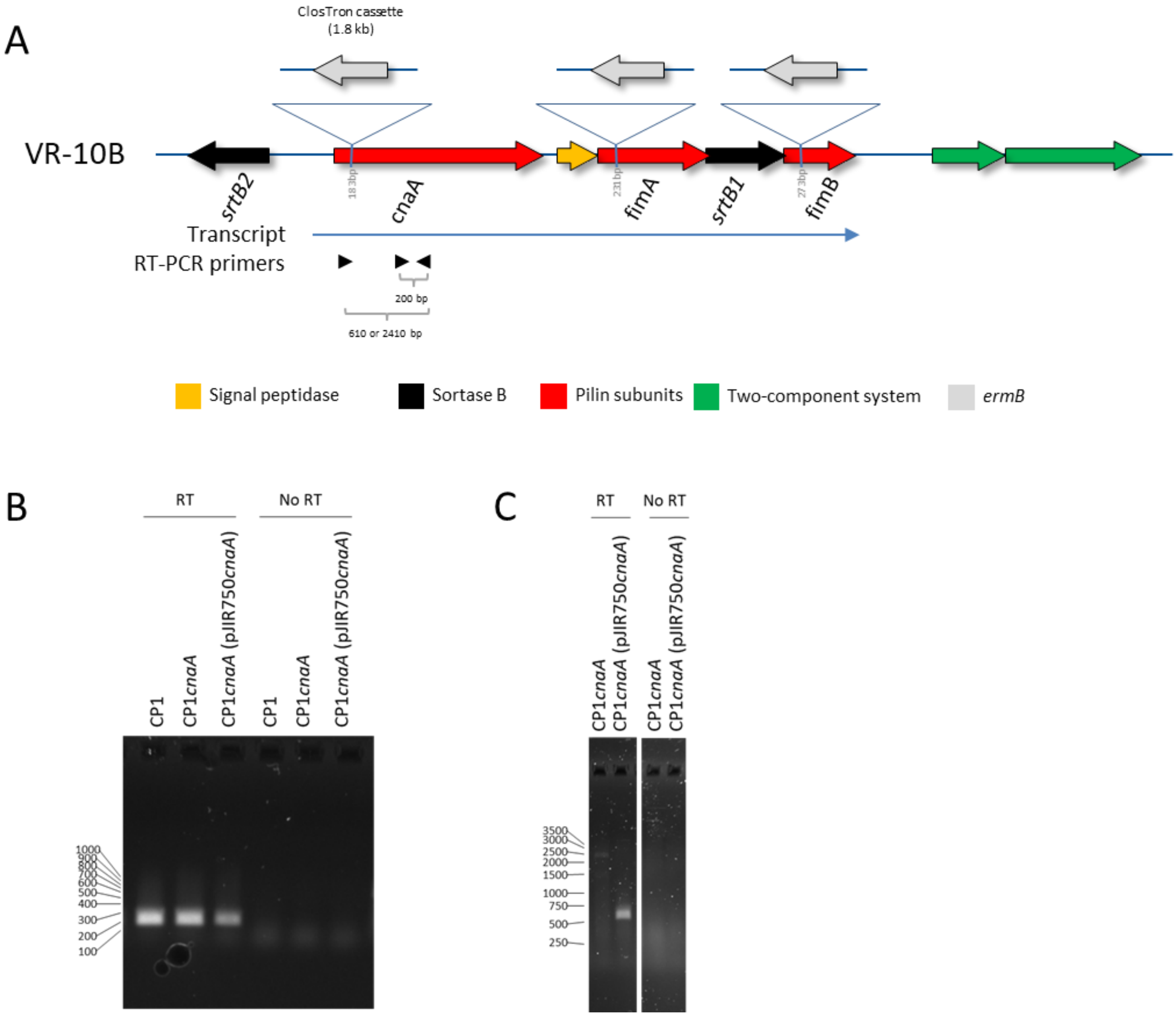

Figure S1. ClosTron mutagenesis and RT-PCR analysis of VR-10B operon. A) Insertion sites for ClosTron cassettes are shown. The blue arrow indicates the predicted polycistronic transcript produced by VR-10B (1), and black arrowheads show location of primers used for RT-PCR, with corresponding amplicon sizes. B and C) PCR was performed on cDNA generated from CP1, CP1*cnaA*, or complement CP1*cnaA (*pJIR750*cnaA*) RNA, using primers B) downstream of (cnaA-F2 and cnaA-R1), or C) flanking the ClosTron insertion site (cnaA-F1 and cnaA-R1). The flanking primers produce a 610 bp product from the native gene, and 2410 bp product if the ClosTron insert is present, as demonstrated in the CP1*cnaA* lane of (C). RT;reverse transcriptase.

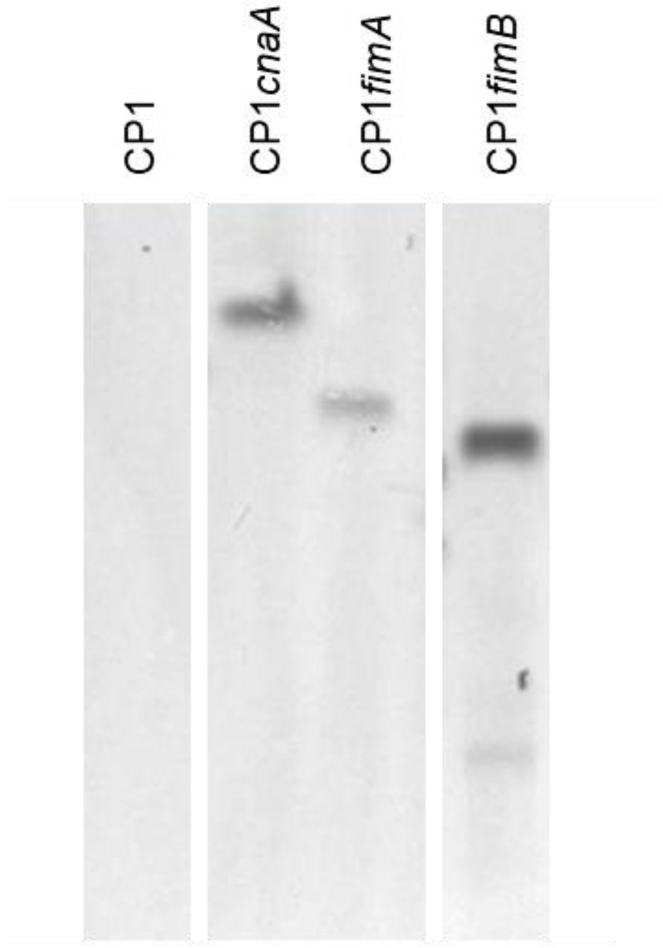

Figure S2. Southern blot analysis of CP1 and isogenic pilin-null mutants. Total genomic DNA from CP1 wild type and isogenic mutant strains was digested with *Dra*I and separated on a 1% agarose gel. Southern blot analysis was performed with a Dig-labelled probe specific for the ClosTron insert using the Dig Luminescent Detection Kit.

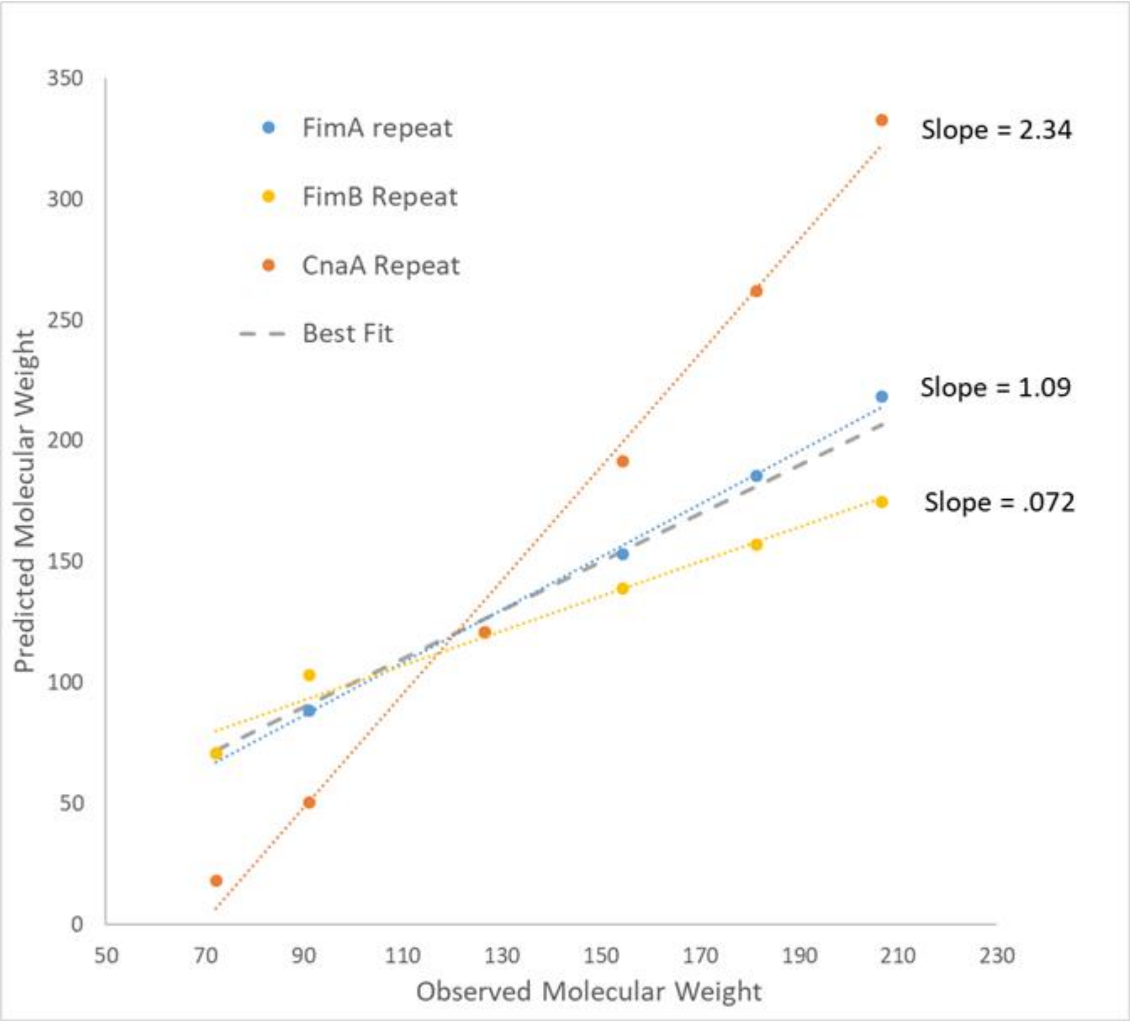

Figure S3. Correlation between pilus band sizes and models of pilus polymer configurations.

Table S1. Summary of variants detected in CP1 pilin-null mutant strains by high-throughput sequencing.

Variant category	CP1cnaA	CP1fimA	CP1fimB
SNP	41	28	23
INDEL	0	0	0
Missense	35	26	20
Nonsense	5	2	3
Start lost	1	0	0
Genes affected	41	26	23

Table S2. Variants detected in CP1 pilin-null mutant strains by high-throughput sequencing and predicted impacts.

Genome position	Strain(s)	Variant Type	Variant (DNA)	Variant (protein)	GeneID	Gene name	Gene Product	Comments
223928	CP1cnaA	missense_variant	c.979G>T	p.Ala327Ser	CP1_00197	cggR	Central glycolytic genes regulator	
229939	CP1cnaA	missense_variant	c.624T>G	p.Asn208Lys	CP1_00202		hypothetical protein	
366530	CP1cnaA	missense_variant	c.861T>G	p.Asp287Glu	CP1_00330	spoIIIE	DNA translocase SpoIIIE	
471737	CP1cnaA	missense_variant	c.2272G>T	p.Gly758Cys	CP1_00432	alaS_1	Alanine--tRNA ligase	
506290	CP1cnaA	missense_variant	c.294G>T	p.Met98Ile	CP1_00459	mepA_3	Multidrug export protein MepA	
749741	CP1cnaA	missense_variant	c.371G>T	p.Gly124Val	CP1_00708	purM	Phosphoribosylformylglycinamidine cyclo-ligase	
844601	CP1cnaA	missense_variant	c.149C>A	p.Thr50Asn	CP1_00788	yjjG	Pyrimidine 5'-nucleotidase YjjG	
937698	CP1cnaA	missense_variant	c.1906G>T	p.Gly636Cys	CP1_00883		hypothetical protein	
1095787	CP1cnaA	start_lost	c.3G>T	p.Met1?	CP1_01028	divIB	Cell division protein DivIB	
1104480	CP1cnaA	missense_variant	c.403G>T	p.Ala135Ser	CP1_01035	rsmH	Ribosomal RNA small subunit methyltransferase H	
1217913	CP1cnaA	missense_variant	c.446C>A	p.Thr149Asn	CP1_01134	ponA	Penicillin-binding protein 1A/1B	
1327915	CP1cnaA	missense_variant	c.358G>T	p.Asp120Tyr	CP1_01238	mbl	MreB-like protein	
1394349	CP1cnaA	missense_variant	c.149C>A	p.Ala50Asp	CP1_01301		hypothetical protein	
1417022	CP1cnaA	missense_variant	c.1047G>T	p.Glu349Asp	CP1_01321		hypothetical protein	
1428204	CP1cnaA	missense_variant	c.473C>A	p.Ala158Glu	CP1_01330	glpF_2	Glycerol uptake facilitator protein	
1461031	CP1cnaA	missense_variant	c.142G>T	p.Asp48Tyr	CP1_01359		hypothetical protein	
1476135	CP1cnaA	stop_gained	c.557C>A	p.Ser186*	CP1_01373	apbC	Iron-sulfur cluster carrier protein	Iron-sulfur cluster carrier protein
1499891	CP1cnaA	missense_variant	c.626G>T	p.Gly209Val	CP1_01393	purR_1	Pur operon repressor	
1617764	CP1cnaA	missense_variant	c.667G>T	p.Ala223Ser	CP1_01505	tldD	Metalloprotease TldD	
1634937	CP1cnaA	missense_variant	c.432G>T	p.Glu144Asp	CP1_01519	carE_1	Caffeyl-CoA reductase-Etf complex subunit CarE	
1685840	CP1cnaA	missense_variant	c.80C>T	p.Pro27Leu	CP1_01565	cbf2	Putative peptidyl-prolyl cis-trans isomerase Cbf2	
1741902	CP1cnaA	missense_variant	c.473G>T	p.Ser158Ile	CP1_01617		IS200/IS605 family transposase ISCpe4	
1897329	CP1cnaA	missense_variant	c.319G>T	p.Asp107Tyr	CP1_01769	thrS	Threonine--tRNA ligase 1	
1988696	CP1cnaA	missense_variant	c.230C>A	p.Ala77Glu	CP1_01858		hypothetical protein	
1990426	CP1cnaA	stop_gained	c.145G>T	p.Glu49*	CP1_01860	epsD	Putative glycosyltransferase EpsD	, Putative glycosyltransferase EpsD
2316840	CP1cnaA	missense_variant	c.1685G>T	p.Arg562Ile	CP1_02192	lon_1	Lon protease	
2436310	CP1cnaA	stop_gained	c.541G>T	p.Glu181*	CP1_02303		hypothetical protein	, hypothetical protein
2479507	CP1cnaA	stop_gained	c.442G>T	p.Glu148*	CP1_02341	pbsA1	Heme oxygenase 1	, hypothetical protein, Heme oxygenase 1
2696726	CP1cnaA	missense_variant	c.1035C>A	p.Ser345Arg	CP1_02521	serS	Serine--tRNA ligase	
2938978	CP1cnaA	missense_variant	c.1771G>T	p.Gly591Cys	CP1_02781		hypothetical protein	
3025656	CP1cnaA	missense_variant	c.68C>A	p.Ala23Asp	CP1_02883	mrnC	Mini-ribonuclease 3	
3181058	CP1cnaA	missense_variant	c.13C>A	p.Gln5Lys	CP1_03037		hypothetical protein	
3236211	CP1cnaA	stop_gained	c.1531G>T	p.Glu511*	CP1_03080	smc_6	Chromosome partition protein Smc	, Chromosome partition protein Smc
3516899	CP1cnaA	missense_variant	c.647G>T	p.Arg216Ile	CP1_03354	endo I	Chitodextrinase	Not present in all reads
744959	CP1cnaA, CP1fimA	missense_variant	c.2354T>G	p.Val785Gly	CP1_00704	purL	Phosphoribosylformylglycinamidine synthase	present in CP1fimB, but low coverage
977744	CP1cnaA, CP1fimA	missense_variant	c.309A>C	p.Leu103Phe	CP1_00914		hypothetical protein	present in CP1fimB, but low coverage
1933104	CP1cnaA, CP1fimA, CP1fimB	missense_variant	c.515T>C	p.Val172Ala	CP1_01808		hypothetical protein	
2307254	CP1cnaA, CP1fimA, CP1fimB	missense_variant	c.709T>G	p.Tyr237Asp	CP1_02181	sdhA_2	L-serine dehydratase%2C alpha chain	
1828419	CP1cnaA, CP1fimB	missense_variant	c.156T>G	p.Asn52Lys	CP1_01711	infA	Translation initiation factor IF-1	present in CP1fimA, but low coverage
2826560	CP1cnaA, CP1fimB	missense_variant	c.355T>G	p.Ser119Ala	CP1_02659		hypothetical protein	present in CP1fimA, but low coverage
3663953	CP1cnaA, CP1fimB	missense_variant	c.221T>G	p.Ile74Arg	CP1_03518		hypothetical protein	present in CP1fimA, but low coverage
311091	CP1fimA	missense_variant	c.2249C>A	p.Ser750Tyr	CP1_00278	rapA	RNA polymerase-associated protein RapA	
539229	CP1fimA	missense_variant	c.3566G>T	p.Gly1189Val	CP1_00482		hypothetical protein	
541127	CP1fimA	missense_variant	c.5464G>T	p.Ala1822Ser	CP1_00482		hypothetical protein	
865326	CP1fimA	missense_variant	c.28G>T	p.Asp10Tyr	CP1_00805	crp	CRP-like cAMP-activated global transcriptional regulator	
936643	CP1fimA	stop_gained	c.851C>A	p.Ser284*	CP1_00883		hypothetical protein	
1576215	CP1fimA	missense_variant	c.152G>T	p.Arg51Ile	CP1_01472		hypothetical protein	
1943361	CP1fimA	missense_variant	c.1349C>A	p.Thr450Asn	CP1_01818	htpG	Chaperone protein HtpG	
2179788	CP1fimA	missense_variant	c.415G>A	p.Gly139Arg	CP1_02032		hypothetical protein	
2269290	CP1fimA	stop_gained	c.196G>T	p.Gly66*	CP1_02132		hypothetical protein	, hypothetical protein
2300924	CP1fimA	missense_variant	c.514C>A	p.His172Asn	CP1_02174		hypothetical protein	
2370652	CP1fimA	missense_variant	c.1205G>T	p.Trp402Leu	CP1_02248	brnQ_2	Branched-chain amino acid transport system 2 carrier protein	
2460078	CP1fimA	missense_variant	c.403C>A	p.Gln135Lys	CP1_02325		Arylsulfatase	
2611080	CP1fimA	missense_variant	c.232G>T	p.Val78Leu	CP1_02449	pfo	Perfringolysin O	
2686249	CP1fimA	missense_variant	c.852G>T	p.Met284Ile	CP1_02512	fadF	putative iron-sulfur-binding oxidoreductase FadF	
2869562	CP1fimA	missense_variant	c.549G>T	p.Gln183His	CP1_02696		hypothetical protein	
3049319	CP1fimA	missense_variant	c.324G>T	p.Lys108Asn	CP1_02907	yaaQ	putative protein YaaQ	
3229040	CP1fimA	missense_variant	c.531G>T	p.Glu177Asp	CP1_03077		hypothetical protein	
3248369	CP1fimA	missense_variant	c.672G>T	p.Lys224Asn	CP1_03093		hypothetical protein	
3329164	CP1fimA	missense_variant	c.636G>T	p.Met212Ile	CP1_03179		hypothetical protein	
3484275	CP1fimA	missense_variant	c.677C>A	p.Ser226Tyr	CP1_03323	araQ_3	L-arabinose transport system permease protein AraQ	
3588138	CP1fimA	missense_variant	c.630G>T	p.Lys210Asn	CP1_03441		hypothetical protein	
3686403	CP1fimA	missense_variant	c.392T>C	p.Ile131Thr	CP1_03557		hypothetical protein	present in some reads of all strains, mapping issue?
3686744	CP1fimA	missense_variant	c.51G>C	p.Arg17Ser	CP1_03557		hypothetical protein	present in some reads of all strains, mapping issue?
3692188	CP1fimA	missense_variant	c.337G>A	p.Ala113Thr	CP1_03567		hypothetical protein	present in some reads of all strains, mapping issue?
527667	CP1fimB	missense_variant	c.711G>T	p.Glu237Asp	CP1_00475	cdaR_1	Carbohydrate diacid regulator	
790989	CP1fimB	missense_variant	c.802C>A	p.Gln268Lys	CP1_00743		hypothetical protein	
1206989	CP1fimB	missense_variant	c.124G>T	p.Val42Leu	CP1_01123	yfkN_2	Trifunctional nucleotide phosphoesterase protein YfkN	
1432197	CP1fimB	missense_variant	c.26G>T	p.Gly9Val	CP1_01334		Ferredoxin--NADP reductase	
1530495	CP1fimB	stop_gained	c.97G>T	p.Glu33*	CP1_01425	greA_2	Transcription elongation factor GreA	, Transcription elongation factor GreA
1821567	CP1fimB	missense_variant	c.98G>T	p.Ser33Ile	CP1_01698	rplX	50S ribosomal protein L24	
1830822	CP1fimB	missense_variant	c.472G>T	p.Asp158Tyr	CP1_01716	rpoA	DNA-directed RNA polymerase subunit alpha	
1974667	CP1fimB	missense_variant	c.20C>A	p.Ala7Glu	CP1_01845		hypothetical protein	
1996460	CP1fimB	stop_gained	c.278C>A	p.Ser93*	CP1_01865	rmlC	dTDP-4-dehydrorhamnose 3%2C5-epimerase	, dTDP-4-dehydrorhamnose 3%2C5-epimerase
2012903	CP1fimB	stop_gained	c.520G>T	p.Glu174*	CP1_01878	agaA_1	Alpha-galactosidase AgaA	, dTDP-4-dehydrorhamnose 3%2C5-epimerase, Alpha-
2269658	CP1fimB	missense_variant	c.623G>T	p.Arg208Ile	CP1_02133		hypothetical protein	
2416590	CP1fimB	missense_variant	c.214G>T	p.Val72Phe	CP1_02281	sipT	Signal peptidase I T	
2704922	CP1fimB	missense_variant	c.916G>T	p.Asp306Tyr	CP1_02532	addA_2	ATP-dependent helicase/nuclease subunit A	
2739558	CP1fimB	missense_variant	c.455G>T	p.Arg152Ile	CP1_02568		Hydroxypyruvate reductase	
3030463	CP1fimB	missense_variant	c.857G>T	p.Gly286Val	CP1_02887	yacL	putative PIN and TRAM-domain containing protein YacL	
3120805	CP1fimB	missense_variant	c.521C>A	p.Thr174Lys	CP1_02974		hypothetical protein	
3343473	CP1fimB	missense_variant	c.1643C>A	p.Ala548Glu	CP1_03190	glgP_2	Glycogen phosphorylase	
3587992	CP1fimB	missense_variant	c.484C>T	p.Pro162Ser	CP1_03441		hypothetical protein	Not present in all reads

1. Wade B, Keyburn AL, Haring V, Ford M, Rood JI, Moore RJ. 2016. The adherent abilities of *Clostridium perfringens* strains are critical for the pathogenesis of avian necrotic enteritis. Vet Microbiol 197:53-61.
